# Where Does N^ε^-Trimethyllysine for the Carnitine Biosynthesis in Mammals Come from?

**DOI:** 10.1371/journal.pone.0084589

**Published:** 2014-01-13

**Authors:** Luigi Servillo, Alfonso Giovane, Domenico Cautela, Domenico Castaldo, Maria Luisa Balestrieri

**Affiliations:** 1 Department of Biochemistry, Biophysics and General Pathology, Second University of Naples, Naples, Italy; 2 Stazione Sperimentale per le Industrie delle Essenze e dei Derivati dagli Agrumi (SSEA), Reggio Calabria, Italy; 3 Dipartimento di Ingegneria Industriale e ProdAl scarl, Università degli Studi di Salerno, Fisciano (SA), Italy; National Institute for Medical Research, Medical Research Council, London, United Kingdom

## Abstract

N^ε^-trimethyllysine (TML) is a non-protein amino acid which takes part in the biosynthesis of carnitine. In mammals, the breakdown of endogenous proteins containing TML residues is recognized as starting point for the carnitine biosynthesis. Here, we document that one of the main sources of TML could be the vegetables which represent an important part of daily alimentation for most mammals. A HPLC-ESI-MS/MS method, which we previously developed for the analysis of N^G^-methylarginines, was utilized to quantitate TML in numerous vegetables. We report that TML, believed to be rather rare in plants as free amino acid, is, instead, ubiquitous in them and at not negligible levels. The occurrence of TML has been also confirmed in some vegetables by a HPLC method with fluorescence detection. Our results establish that TML can be introduced as free amino acid in conspicuous amounts from vegetables. The current opinion is that mammals utilize the breakdown of their endogenous proteins containing TML residues as starting point for carnitine biosynthesis. However, our finding raises the question of whether a tortuous and energy expensive route as the one of TML formation from the breakdown of endogenous proteins is really preferred when the substance is so easily available in vegetable foods. On the basis of this result, it must be taken into account that in mammals TML might be mainly introduced by diet. However, when the alimentary intake becomes insufficient, as during starvation, it might be supplied by endogenous protein breakdown.

## Introduction

N^ε^-trimethyllysine (TML) is a non-protein amino acid which has an important role as metabolic intermediate being recognized as the precursor of carnitine, a metabolite essential for the fatty acid transport and utilization in mitochondria. Carnitine is biosynthesized through a sequence of four enzyme catalyzed reactions. In the first reaction, catalyzed by the N^ε^-trimethyllysine hydroxylase, TML is converted into 3-hydroxy-TML (HTML). Then, a specific aldolase cleaves HTML into glycine and 4-N-trimethylaminobutyraldehyde, which is successively oxidized to 4-N-trimethylaminobutyrate. Finally, the hydroxylation of the 4-N-trimethylaminobutyrate produces 3-hydroxy-4-N-trimethylaminobutyrate, which is better known as carnitine [1]. In some animals, especially carnivores, carnitine is largely introduced with the diet and a recent study, which stimulated a considerable debate in the scientific community, suggested the possibility that the intestinal metabolism of exogenous carnitine could promote atherosclerosis in humans [2]. However, it is recognized that mammals synthesize carnitine endogenously and, to date, no biosynthetic route alternative to that reported above is known. The biosynthesis of TML has been a puzzling biochemical problem for long time. In fact, attempts aimed to demonstrate in higher organisms its formation from lysine methylation have been unsuccessful so far. Actually, the presence of an enzyme which methylates lysine by using S-adenosylmethionine as a methyl donor and forming N**^ε^-**trimethyllysine was reported only in the mould *Neurospora crassa* [3]. Since then, neither other reports in the scientific literature describing a similar enzyme in some other organism nor more information on the *Neurospora crassa* enzyme nor its cloning has appeared.

To date, the generally accepted view is that the metabolic source of TML is the hydrolysis of proteins which contain TML as a post-translational modification of some lysine residues. It is well known, in fact, that in mammals proteins such as calmodulin, histones, cytochrome c, and myosin contain TML residues [4], [5]. Therefore, it is conceivable that free TML may be released in the course of the protein turnover and, then, utilized for carnitine production, although a direct evidence of this formation route has never been reported. Indeed, only experiments, conducted on exogenous proteins, chemically radiolabeled in vitro by the incorporation of [^3^H]methyl groups into lysine residues and then perfused into animal livers, showed that free TML was released [6]–[8]. On the other hand, it would have been difficult to imagine another origin for TML, as this amino acid is believed to be rather rare in its free form. Actually, in the natural world, besides animals, there are few other sources known to contain free TML. As matter of fact, the occurrence at fair level of TML, as a free amino acid, has been reported in seaweeds, especially those of the Laminaria genus. For this reason, TML has also the trivial name of laminine [9], [10].

Recently, we showed for the first time the occurrence of N^G^-methylated derivatives of arginine in the most important vegetables utilized for human nutrition [11]. Intriguingly, also these compounds are believed to be formed in human organism by degradation of proteins containing N^G^-methylated arginine residues arising from post-translational modifications [12]. In the course of this study, we developed a rapid HPLC method to separate, before quantification by ESI-MS/MS, N^G^-methylarginines in short times without need of derivatization. During the analysis of numerous extracts of vegetables important in human nutrition, we invariably noticed in the chromatogram the presence of a peak much more retained and intense than those of arginine N^G^-methylated derivatives. Here, we report the identification of this peak and show that it corresponds to TML, which, therefore, appears to be ubiquitous, not at negligible levels, in all the vegetal species examined.

## Materials and Methods

### Reagents

N^ε^-trimethyllysine, N^G^-monomethylarginine, homoarginine, formic acid, ammonium formate, and the 0.1% solution of formic acid in water used for the LC-ESI-MS analyses were from Sigma-Aldrich (Milan, Italy). The standard mixture of L-amino acids, containing Ala, Arg, Asp, Glu, His, Ileu, Leu, Lys, Met, Phe, Pro, Ser, Thr, Tyr, Val, and Cys at 2.5 mM concentration in 0.01 M HCl, was from Pierce. AccQ FLuor reagent was from Waters (Milan, Italy). Milli-Q water was used for all the preparations of solutions and standards.

### Vegetable sources

Fruits, leafy vegetables, potato (tuber), flours of Gramineae and Leguminosae were purchased in various local supermarkets. Four different lots of 1 kg of each vegetal source were analyzed at various times. Samples of alfalfa (*Medicago sativa*) and nettle (*Urtica dioica*) plants were supplied by the city botanical gardens. All vegetables used are reported in Table 1.

**Table 1:** N^ε^-trimethyllysine (TML) content in some vegetables estimated by LC-ESI-MS/MS.

Samples	TML ranges (mg/Kg)
***Leguminosae flours***	
Soy	1–2
Broad bean	1–3
Chickpea	2–5
***Gramineae flours***	
Wheat	0.5–4
Maize	0.5–1
Rye	0.5–3
***Solanaceae***	
Sweet pepper berry	8–18
Tomato berry	0.8–2.5
Potato tuber	1–4
***Leaf vegetables***	
Lettuce	0.1–1
Spinach	0.5–2
Chicory	0.5–1
Nettle	0.5–2
Alfalfa	2–10
***Fruits***	
Kiwi	0.2–0.9
Banana	0.4–1.1
Apple	0.1–0.2
Pear	0.1–0.2
***Fruit Juices***	
Grapefruit	0.4–0.9
Lemon	0.1–0.6
Mandarin	0.1–0.4
Bergamot	0.4–0.8
Orange	0.4–0.7
Pineapple	0.5–0.9
Grape	0.5–0.9

Ranges were obtained by analyzing four lots of each sample in duplicate.

### Sample preparations for TML determinations by LC-ESI-MS/MS

Citrus fruit juices were prepared by hand squeezing the fruits. Juices were previously centrifuged at 12000× g for 15 min and then the supernatants were diluted 1∶1 with a 0.2% solution of formic acid in water. As for flours and leafy vegetables, the samples were homogenized in a mixer with formic acid 0.2% v/v in 1∶3 (w/w) ratio, whereas, for fruits, the edible part (200 g) was homogenized in a mixer with formic acid 0.2% v/v in 1∶1 (w/w) ratio. The homogenates were kept for 2 h under constant agitation and then centrifuged at 18000× g for 30 min at 4°C. The supernatants were finally frozen and kept at −20°C until used for analytical determinations.

### Determination of TML by LC with ESI-MS/MS detection

The LC-ESI-MS analyses were performed with HPLC Agilent 1100 series equipped with on line degasser and automatic injector coupled on-line with an Agilent LC-MSD SL quadrupole ion trap. The MS acquisition was performed by ESI in positive ion mode with nitrogen as the nebulising and drying gas under the following conditions: nebulizer pressure, 30 psi; drying temperature, 350°C; drying gas, 7 L/min. The Ion Charge Control (ICC) was applied with target set at 30000 and maximum accumulation time at 20 ms. The measurements were performed from the peak area of the Extracted Ion Chromatogram (EIC). The quantification was achieved by comparison with the calibration curves obtained with standard solutions. The standard stock solution of TML was prepared at a concentration of 200 mg/L and additional calibration levels (25, 5, 2, 1 and 0.1 mg/L) were prepared by serial dilution with water containing 0.1% formic acid and stored at 4°C. The calibration curve was built using these standard solutions. The response of the MS/MS detection follows a linear calibration curve between 0.1 and 25 mg/L with a correlation coefficient of 0.99. The optimization of the instrumental parameters for the analyses of TML was performed by continuous infusion of 5 µM standard solution in 0.1% formic acid. The mass cut-off and the fragmentation amplitude were optimised in order to obtain the most efficient MS/MS transitions from the positively charged precursor ion [M+H^+^] to the fragment ions. The more intense 189.2→130 MS/MS transition was used for TML quantification. The 189.2→84 and m/z 189.2→60 MS/MS transitions were utilized to confirm TML identification. Successively, volumes of 10–20 µL of standard solutions or samples were analyzed by HPLC-ESI-MS/MS by using the silica column Supelcosil™ LC-Si 3.3 cm×4.6 mm i.d., 3 µm particle size. The elution was performed isocratically at a flow rate of 100 µL/min by utilizing 75% of Sol. A (0.1% formic acid in water) and 25% of Sol. B (100 mM ammonium formate in water titrated to pH 4.5 with formic acid).

The retention times and peak areas of the monitored fragment ions were determined by the Agilent software Chemstation version 4.2. Recovery of TML from the various matrices was determined by analyzing portions (10 g) of homogenized samples. Portions were spiked with a know amount of TML in formic acid 0.1%. The standard solutions added to the samples were 2 µmol/L. Samples of the same homogenized matrix without the addition of TML were also analyzed. The recovery of TML was obtained separately. Percentage recoveries were based on the difference between the total amount in the spiked samples versus that in the unspiked samples. Reproducibility was assesses by the determination of recovery of six individual samples. The limit of detection of TML was assessed by utilizing only the standard solutions in formic acid 0.1%. The limit of detection was determined as the concentration of TML which gave a peak height three times that of the background noise.

### Determination of TML and amino acids by LC with fluorescence detection

TML quantification in the samples was also performed by reverse-phase HPLC employing a Waters instrument mod. 2690 equipped with the fluorescence detector mod. 474. The juice or extract samples of about 10 mL were centrifuged at 12000× g at 4°C for 10 min. Then, 5 mL of supernatant was filtered and loaded on a column (5×1 cm) filled with Bio-Rad AG 50WX8-(H^+^) resin. After loading, the column was washed with five volumes of Milli-Q water and, then, the amino acids were one-step eluted with 10 mL of 15% ammonia solution in water. In order to exhaustively remove ammonia which heavily interfered with TML determination, after drying the eluate in a rotavapor, the residue was dissolved in 2 mL of NaOH 0.01 M in water and then vacuum dried again. The residue was finally dissolved in 2 mL of 0.01 M HCl in water and the solution filtered through a 0.45 µm filter. The derivatization of TML in the samples was accomplished with AccQ (6-aminoquinolyl-N-hydroxysuccinimidyl carbamate), which is usually employed in the quality control of fruit juices for the determination of the free amino acids by HPLC with fluorescence detection [13]. AccQ-N**^ε^-**trimethyllysine was fluorimetrically detected by excitation at 350 nm and emission at 395 nm. AccQ-N**^ε^-**trimethyllysine was identified on the basis of the retention time and quantified by comparison of the sample peak area with the calibration curve.

## Results

### Determination of TML in vegetable sources

**Figure 1:**
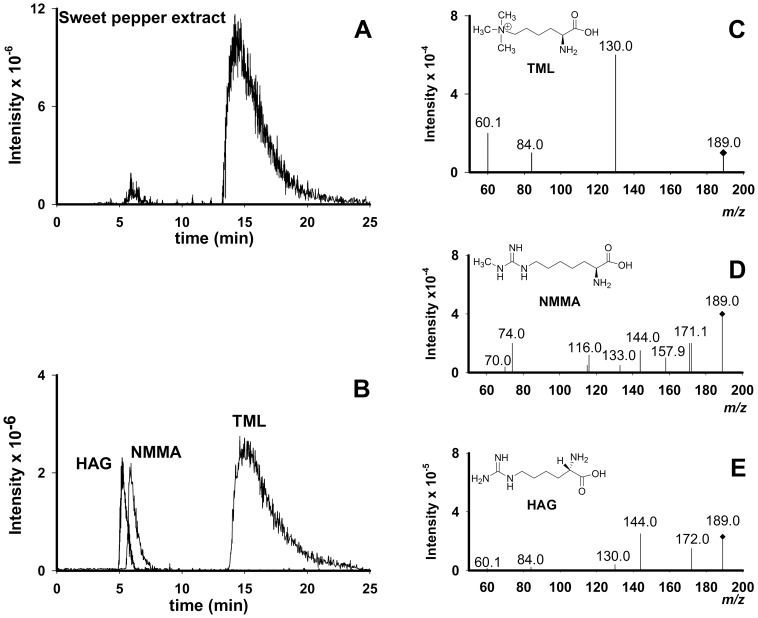
Chromatographic analysis of a sweet pepper berry extract. The elution was performed isocratically on the silica column Supelcosil™ LC-Si by utilizing 75% of Sol. A (0.1% formic acid in water) and 25% of Sol. B (100 mM ammonium formate in water titrated to pH 4.5 with formic acid). (**A**). The MS/MS extracted ion chromatograms obtained from the sweet pepper berry sample. The transitions followed were 189.2→144, 189.2→74 (peak at r.t. 6.0 min), and 189.2→130 (peak at r.t. 15 min). Homoarginine, followed by the transition 189.2→144, was not detected in the sample. (**B**). The MS/MS extracted ion chromatograms of a standard mixture of homoarginine (HAG), N^G^-methylarginine (NMMA) and N**^ε^-**trimethyllysine (TML). The transitions followed were 189.2→144 (specific for HAG, peak at r.t. 5.5 min), 189.2→74 (specific for NMMA, peak at r.t. 6.0 min), and 189.2→130. The transition 189.2→130 is common for both HAG and TML (peak at 15.0 min). (**C**), (**D**), (**E**). Fragmentation patterns of the three peaks from the standard mixture of Panel B. The fragmentation pattern of the peak at r.t. 15 min (Panel A) being identical to that of the standard TML was not reported.

The quantification of TML in the vegetal matrices was performed by the same chromatographic procedure that was advantageously employed to analyze N^G^-methylated arginine derivatives in vegetables [11]. This procedure has proven to be suitable for polar substance analysis with ESI-MS/MS detection and does not require sample derivatization. The chromatography was performed with a short silica column (Supelcosil™ LC-Si), employing isocratic elution conditions. When analyzing the content of methylarginines in vegetables, multiple reaction monitoring was used for the analyte quantification. The transition utilized for N^G^-monomethylarginine (NMMA) MS^2^ quantification was 189.2→74. Incidentally, the m/z of protonated TML is 189.2 too. In all vegetable samples, this lucky coincidence allowed the observation of an intense peak in the total ion chromatogram (TIC), emerging at a retention time (r.t.) of about 15 min, much more retained than that of NMMA. As an example, the MS^2^ total ion chromatogram (TIC) by isolating at m/z 189.2 from a sample of sweet pepper berry extract is reported (Figure 1, panel A). The MS^2^ fragmentation pattern of the peak at r.t. 15 min (Figure 1, panel A) shows only three fragments at m/z 130, 84, 60 (Figure 1 panel C) which are typical of N^ε^-trimethyllysine MS^2^ fragmentation [14], as also confirmed by comparison with the fragmentation pattern of the TML standard solution. The smaller peak at r.t. 6.0 min (Figure 1, panel A) corresponds to N^G^-monomethylarginine. Anyway, besides the identity of the MS^2^ fragmentation pattern with that of TML standard solution, the identification of the unknown peak was also confirmed by the identity of its retention time with that of an authentic TML standard solution (Figure 1, panel B). Moreover, when changing the chromatographic conditions by varying the ammonium formate concentration in the eluent, it was found that the retention time of TML on the LC-Si column strongly varied depending on the concentration of ammonium formate in the eluent, as also observed for methylarginines [11]. Therefore, in order to further confirm the peak identity, various eluent composition changes were tried. In each case, the retention times of the standard TML peak and that of the unknown compound varied in the same way. However, besides NMMA, TML is also isobaric with homoarginine (HAG), a substance also reported to occur in some vegetables such as grass pea (*Lathyrus sativus*) [15] and lentils [16]. In order to exclude the possibility to mistake homoarginine for TML, we run HAG standard solution in the same chromatographic conditions. We found that HAG shows a much shorter retention time (about 5.5 min) than TML, thus the two compounds are completely resolved in the chromatographic conditions we utilized (Figure 1, panel B). This is important as the fragments at m/z 130, 84 and 60 present in MS/MS fragmentation pattern of TML also occur in the MS/MS fragmentation pattern of homoarginine, besides other fragments (Figure 1, panel E). Therefore, a complete chromatographic resolution is mandatory for a reliable attribution and quantification when both compounds are present in the same sample. Our results show that TML is present in all the vegetables analyzed (Table 1), as demonstrated for each vegetable source by the comparison of retention times and MS^2^ fragmentation patterns with the authentic standard. In particular, the highest concentration was found in sweet pepper fruit. It is interesting to note that fruit juices, largely utilized in human nutrition, such those of orange and grapefruit, also contain levels of TML in the same range of the content of some essential amino acids, such as lysine and leucine [17]–[22]. Also, it is of interest the observation that one of the highest level of TML was found in leaves of alfalfa, a plant of paramount importance for the cattle feeding.

### Evaluation of the percentage recoveries

In order to check the performance of the analytical procedure, the percentage recoveries, the reproducibility and the limits of detection for TML were assessed. Recoveries of TML, determined as reported in Materials and Methods section, were in each case higher than 95% for all analyzed samples. The reproducibility, assessed by calculating the standard deviation of six recoveries, resulted less than 5%. The detection limit, determined as that concentration of TML which gave a peak height three times that of the background noise, was 10 nmol/L.

**Figure 2:**
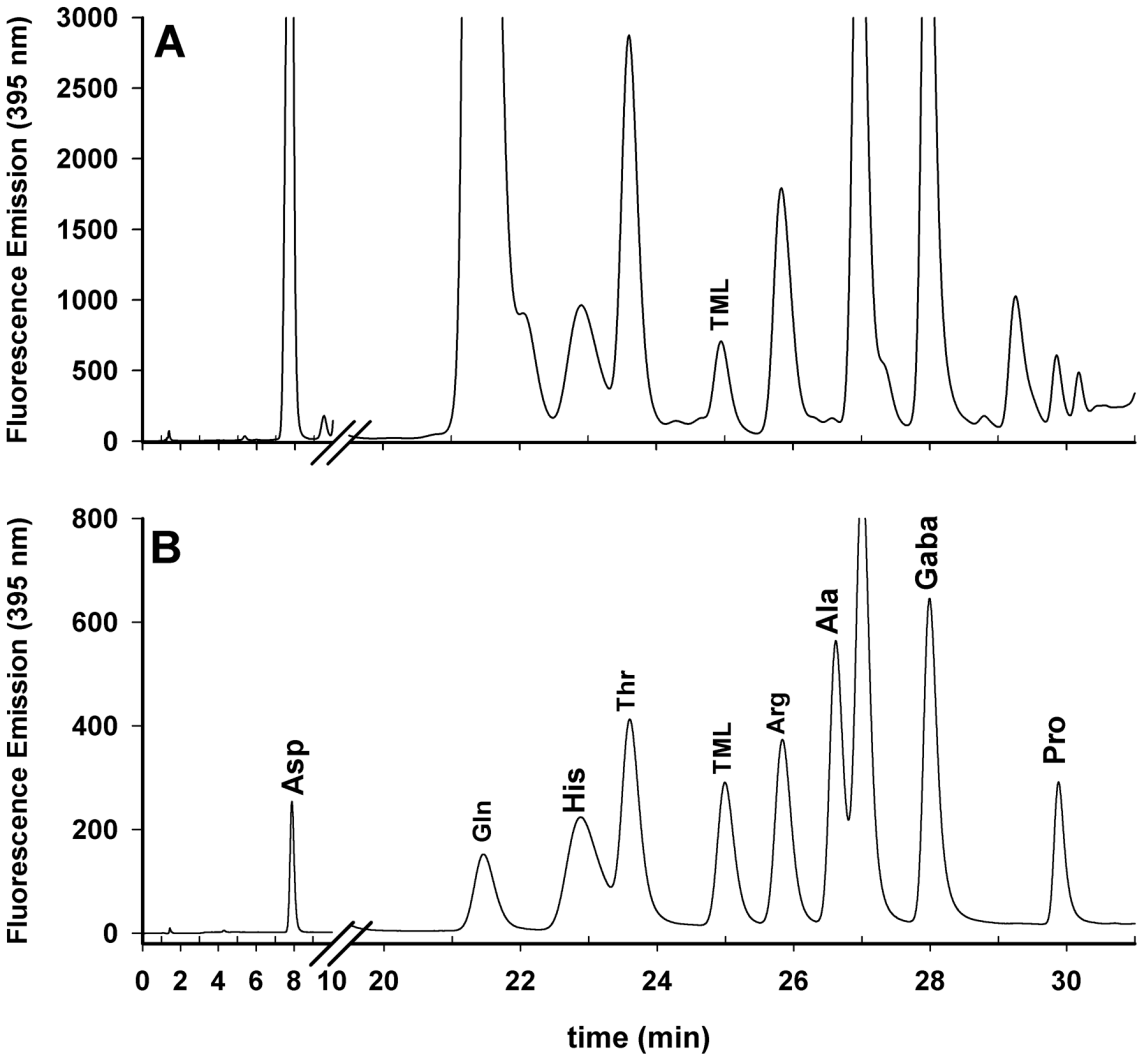
RP-HPLC chromatography with fluorescence detection. Chromatography of the TML content in a sweet pepper berry extract (**A**). Chromatography of amino acid standard mixture added with N**^ε^-**trimethyllysine (**B**). Details of chromatography and sample preparation are reported in the text.

The presence of TML in the vegetable samples was further confirmed by an independent analytical approach, based on HPLC with fluorescence detection, commonly used to analyze amino acids in food sources [13]. This procedure involves the purification of the vegetal extract by a passage on a short column of AG50WX8 (H+ form). Moreover, this procedure also allowed to have an estimation of the matrix effect in the analyses of some samples by comparing the results obtained by HPLC-ESI-MS/MS with and without the purification passage on the AG50WX8 column (see Materials and Methods). In all cases, the samples without the passage on column showed values from 2% to 15% lower than the purified samples. An important aspect of TML analysis by HPLC with fluorescence detection consists in the sample treatment after the passage on the AG50WX8 column. Generally, for amino acid analysis, the column is eluted with a 15% ammonia solution. Afterwards, the eluted solution is vacuum dried and the residue is suspended in a suitable solvent and derivatized. However, we found that in this way a broad peak, due to residual ammonia presence, heavily interfered with determination of derivatized TML, as both substances emerged in the same time range. Instead, the treatment with a diluted solution of sodium hydroxide, as described in Materials and Methods, exhaustively removed ammonia from the dried samples and completely eliminated such interference (Figure 2). Results obtained by HPLC-ESI-MS/MS and by HPLC with fluorescence detection were also compared for tomato berry, alfalfa leaves and sweet pepper berry extracts (Figure 2). The HPLC analyses confirmed the presence of TML in these vegetables at levels comparable within +/−10% to those obtained by HPLC-ESI-MS/MS.

## Discussion

In this paper we reported the occurrence of TML in a consistent number of vegetables and vegetable derived products. Specifically, in the samples examined, free TML levels were in the same range as other free amino acids [17]–[24]. This result is of noticeable interest as the presence of free TML in vegetables, excepting seaweeds, was practically ignored so far. To date, only one study reports unequivocal identification and quantification of free TML in a plant extract [23]. On the contrary, several studies report in some plants the occurrence of proteins containing the TML residue in their primary structure as a post-translational modification of lysine residues. Among these proteins, there are calmodulin from *Papaver somniferum* and *Euphorbia lathyris* [24], [25], and cytochrome c from wheat germ, which has been found to contain two TML residues [26]. Another important protein, owing to its essential role in carbon dioxide fixation in the vegetal cell chloroplasts, is the ribulose-1,5-bisphosphate carboxylase oxygenase (RuBisCo) which, in some plant species, has been found to contain TML in the N-terminal region of its large subunit [27], [28]. Since the biosynthetic pathway for TML formation, as free amino acid, is still unexplored in plants, it cannot be ruled out that the free TML also in plants origins from the breakdown of TML residue containing proteins, as it is supposed for mammals. The role of free TML in all plants we examined can be at the present only hypothesized. It may be supposed that, at least in part, TML is utilized for carnitine biosynthesis, as it happens for mammals. In fact, it has been recently demonstrated that in *Arabidopsis thaliana* the carnitine biosynthetic pathway shares similar features with the pathway of mammals and fungi [23]. An other possible role of TML could be that to be part of the pool of osmolytes, as it is hypothesized for seaweeds [10], and thus it could play a stress protective function in the vegetal cells. Anyway, whatever the TML role in plants may be, an important question arises, as a consequence of our results, about the true source of TML in mammals.

**Figure 3:**
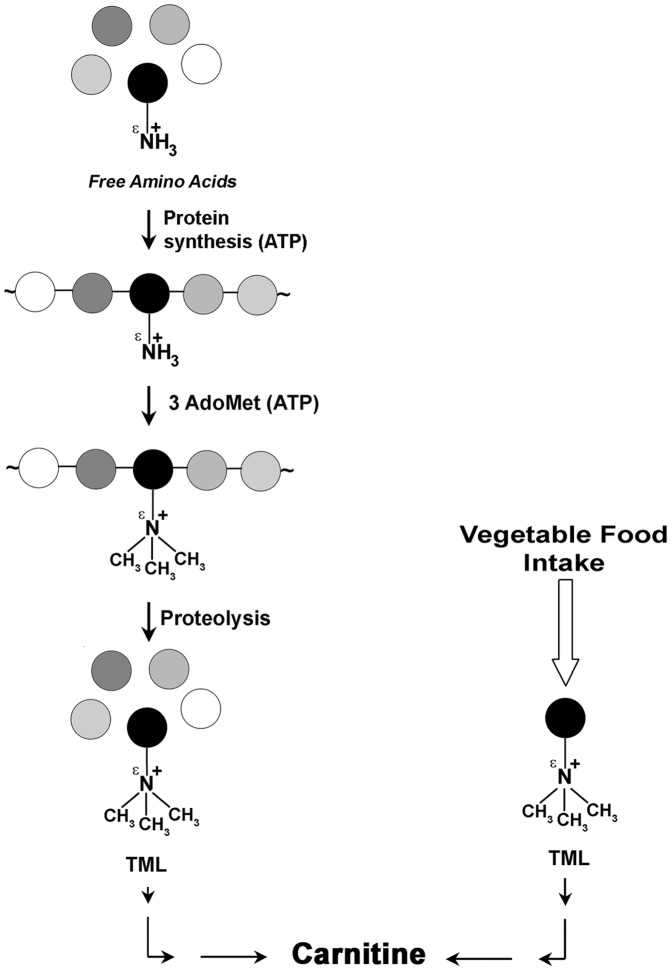
Pathways for the carnitine formation. Left side: The biochemical pathway, according to the view generally accepted, entails, as the metabolic source of TML in mammals, the hydrolysis of proteins which contain TML as a post-translational modification of some lysine residues. Right side: The vegetal food intake, as suggested in this paper, directly provides mammals with the TML to be converted into carnitine. Circles depict amino acids. The black filled circle indicates lysine. AdoMet: S-adenosylmethionine. Both protein and AdoMet syntheses require ATP (indicated in parentheses).

The current opinion is that mammals utilize the breakdown of their endogenous proteins containing TML residues as starting point for carnitine biosynthesis (Figure 3). But is it conceivable to resort to such tortuous and energy expensive route when the substance is so easily available from vegetable foods? This consideration even better applies to herbivorous mammals for which the intake of free TML through foods should be higher than for other mammals. From this point of view, it is of interest our finding that alfalfa leaves, one of the most important forage grass for the cattle feeding, resulted one of the richest source of free TML among the vegetables examined (Table 1). In this respect, besides the consideration that the amount of TML from vegetables assumed by the herbivores seems sufficient for their carnitine biosynthesis, it is also important to point out that rat and guinea pigs orally supplemented with free TML have an increased rate of carnitine biosynthesis and excretion [29]–[31]. Conversely, dietary proteins (casein, soy proteins, and wheat gluten) were found to be poor source of TML [31]. In light of such evidence, our hypothesis seems founded on a firm ground as it has already been proved that animals orally supplemented with free TML showed increased rates of carnitine biosynthesis and excretion [29], [31].

On the other hand, also for humans with the particular dietary habit to eat only vegetable foods (vegetarians or vegans), it is hardly conceivable that only endogenous TML can suit the needs for their carnitine biosynthesis. Indeed, it has been shown that vegetarian adults have slightly reduced plasma carnitine levels compared to omnivorous individuals [32], [33]. However, serum carnitine levels are more markedly depressed in vegetarian children [32] and in premature infants who do not receive a dietary source of carnitine [34]. However, this could be ascribed to immaturity of the biosynthetic conversion machinery of TML into carnitine. Lastly, a warning that could be relevant to vegans is that the assumption of free TML through vegetables consumption may represent a risk factor for those subjects affected by dysregulation of the carnitine biosynthesis due to a mutation of the N^ε^-trimethyllysine hydroxylase gene [35].

In conclusion, although it cannot be excluded that a certain amount of TML might be produced by the hydrolysis of endogenous proteins containing such residue as a posttranslational modification, it does not appear likely that this route might be the preferred one when the substance is so easily available through a vegetable containing diet (Figure 3). Conversely, when the dietary intake become insufficient, it cannot be ruled out that the breakdown of endogenous proteins could become the prime source of TML. From this point of view, it seems reasonable to suggest that in mammals TML utilized for carnitine biosynthesis might be mainly taken from diet (for herbivore animals this should be even more likely). However, when the alimentary intake fails, as in case of starvation, TML might be produced from endogenous protein breakdown, similarly to the essential amino acids when their intake becomes insufficient.
